# Mechanisms of cellular senescence combined with molecular docking strategies: A biomarker study of potential therapeutic targets for allergic rhinitis

**DOI:** 10.1371/journal.pone.0338309

**Published:** 2026-01-09

**Authors:** Qingyong Chen, Han Zhang, Yue Wu, Jixin Liu, Dongqing Wang, Dezhong Sun, Zhipeng Chen, Liqiang Lin, Huaiqing Lv, Qiang Shao

**Affiliations:** 1 Linyi People’s Hospital Affiliated to Shandong Second Medical University, Linyi, China; 2 No.One Clinical Medicine School of Binzhou Medical University, Bing Zhou, China; 3 Junan County People’s Hospital, Linyi, China; Guangdong Nephrotic Drug Engineering Technology Research Center, Institute of Consun Co. for Chinese Medicine in Kidney Diseases, CHINA

## Abstract

**Objective:**

Bioinformatics and molecular docking methods were used to screen potential biomarkers of cellular senescence in allergic rhinitis (Allergic rhinitis AR), which provided a theoretical basis for revealing the mechanism of AR and exploring new therapeutic approaches.

**Methods:**

Four AR-related gene chips (GSE19187, GSE43523, GSE44037, and GSE51392) were downloaded from the gene expression database (GEO) for data pooling. Screening differential genes (DEGs) were taken to intersect with cellular senescence-related genes (SRGs) to obtain differential senescence genes (DESRGs). The differential senescence genes were subjected to Gene Ontology Database (GO) functional analysis, Kyoto Encyclopedia of Genes and Genomes (KEGG) pathway analysis, and GSEA enrichment analysis. Protein-protein interaction (PPI) networks were constructed through the STRING database, MCODE plugin weights were analyzed to identify important gene cluster modules, and Hub genes were screened using the CytoHubba plugin topological network algorithm. Hub gene protein interactions network (GeneMANIA) was constructed by the GeneMANIA database. Predict Hub gene construct mRNA-miNA-lncRNA interactions by miRanda, miRDB, miRWalk, TargetScan, and spongeScan databases; construct Hub gene transcription factor regulatory networks by TRRUST database; analyze Hub gene-drug interactions by DGIdb database and select commonly used drugs in the clinic for molecular docking validation.

**Results:**

A total of 264 differential genes were screened in the training set with corrected P.adj < 0.05 and |log2FC| ≥ 1.2 as the filtering condition, and a total of 866 cellular senescence genes, and 20 differential senescence genes (DESRGs) were obtained by taking the intersection of the two. A total of 19 Hub genes were obtained after PPI analysis, which were CCL2, STAT1, TLR2, IGFBP3, TLR3, KLF4, IL1RN, IRF1, SERPINB2, DPP4, MME, NQO1, SAMHD1, XAF1, PHGDH, EIF4EBP1, CTH, HSPA2, AHR The gene-protein interaction network identified 19 Hub genes associated with 21 functional proteins. 5 of the Hub gene loci were associated with 29 miRNAs and 53 lncRNAs. The transcription factor regulatory network obtained 15 transcription factors capable of regulating Hub genes. The analysis of drug–gene interactions identified 489 drugs that target hub genes. For example, in the case of budesonide, the interacting genes STAT1, TLR2, TLR3, and AHR were selected for molecular docking. Similarly, for mometasone, the interacting genes TLR2 and CTH were chosen for molecular docking.

**Conclusion:**

Mining AR-related Hub senescence genes by bioinformatics analysis, constructing PPI network, ceRNA network, transcription factor regulatory network, gene-drug interaction network and molecular docking validation, we screened 19 CCL2, STAT1, TLR2, IGFBP3, TLR3, KLF4, IL1RN, IRF1, SERPINB2, DPP4, MME, NQO1, SAMHD1, XAF1, PHGDH, EIF4EBP1, CTH, HSPA2, and AHR are expected to be Hub genes for potential diagnostic and therapeutic biomarkers, which will provide targets and new insights for further in-depth explorations of AR cellular senescence-related mechanisms of action and therapy.

## 1. Introduction

Allergic rhinitis AR, also known as allergic rhinitis, is a disease of hyperresponsiveness of the nasal mucosa characterized by symptoms such as sneezing, nasal congestion, itching, and runny nose [1].AR affects people of all ages globally and has a significant impact on patients’ quality of life, productivity, and healthcare costs, while at the same time taking up a large amount of healthcare resources and imposing a heavy economic burden on national governments It also takes up a large amount of healthcare resources and imposes a heavy economic burden on national governments and societies, making it a global public health problem [2]. According to epidemiologic surveys, the incidence of AR is 10% to 40% worldwide and is increasing yearly [3]. At present, there is still no complete cure for AR, and the fundamental reason is that its pathogenesis has not yet been elucidated [4]. Recent studies have shown that cellular senescence is involved in the pathogenesis of AR.

Cellular senescence is a state of permanent cell cycle arrest that involves a variety of biological processes, normal aging, and different diseases [5]. The main features of cellular senescence are irreversible limitations on cell proliferation, cellular stress, telomere shortening, DNA damage, and complex senescence-associated secretory phenotypes (SASPs) [6]. Currently, cellular senescence has been recognized as an important driving mechanism in chronic lung diseases, such as idiopathic pulmonary fibrosis and chronic obstructive pulmonary disease (COPD) [7]. However, little is known about the role of cellular senescence in the pathogenesis of AR. In an allergic asthma study, it was shown that bronchial fibroblasts in asthma patients had reduced telomere length compared to healthy individuals, and this reduction was associated with an increase in the cellular senescence marker β-galactosidase [8]. This study suggests that cellular senescence may also play a key role in AR. Therefore, further understanding the functional role of cellular senescence in the pathogenesis of AR and exploring the senescence characteristics of AR may inform the development of interventions. Nasal glucocorticosteroids are first-line agents for the treatment of AR and belong to class A evidence [9]. Budesonide and mometasone are commonly used nasal corticosteroids in clinical practice. Their strong anti-inflammatory effects can alleviate the clinical symptoms of AR patients by suppressing the release of cytokines and other inflammatory mediators. In this study, molecular docking was performed to validate the interactions of hub genes associated with budesonide and mometasone.

In this study, we screened 19 Hub senescence genes (CCL2, STAT1, TLR2, IGFBP3, TLR3, KLF4, IL1RN, IRF1, SERPINB2, DPP4, MME, NQO1, SAMHD1, XAF1, PHGDH, which are closely related to AR, through bioinformatics combined with several related databases, (EIF4EBP1, CTH, HSPA2, AHR). Functional proteins (PPIs), enrichment pathways, ceRNA networks, and transcription factor regulatory networks associated with AR were predicted based on 19 Hub genes. Meanwhile, the DGIdb database was utilized to search for potential drugs targeting each senescent gene of AR, and molecular docking validation was performed. Taken together, this work may provide new insights and a theoretical basis for studying the potential regulatory mechanisms associated with cellular senescence and the related therapies for AR.

## 2. Information and methods

### 2.1 Data sources and processing

**Fig 1:**
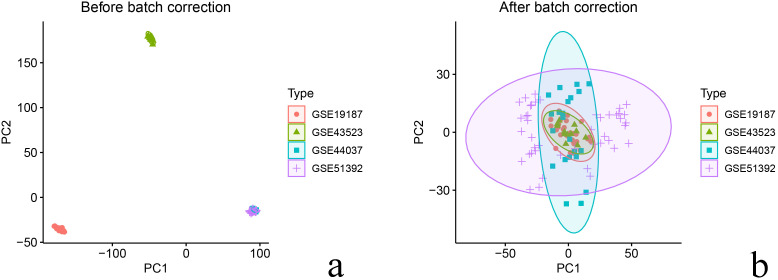
Principal-component analysis (PCA) of the merged GEO datasets. **(a)** Before batch correction, **(b)** After batch correction.

The GEO database was searched for “Allergic rhinitis AR”, and the screening criteria were based on the data type of experimental study, the type of study was expression profile microarray, and the experimental samples were human tissue specimens. A total of 4 sets of gene expression profiling datasets were obtained from the screening, which were GSE19187, GSE43523, GSE44037, and GSE51392, and the 4 sets of datasets were pre-processed with the limma package of R software (version 4.2.2). The four data sets were combined to obtain 52 normal tissue samples (control group) and 58 AR tissue samples (test group). In order to avoid the batch effect caused by the differences in experimental conditions, technical platforms, sample sources, and other factors affecting the reliability and accuracy of the datasets, this study first used the normalizeBetweenArrays function to standardize the differences between different groups of the four datasets, removing the differences between groups to make the comparison between the samples more accurate and reliable; and then used the R software sva program package for the combined datasets to obtain the normal tissue samples of 52 cases (control group) and AR tissue samples of 58 cases (experimental group). The data were corrected by ComBat batch correction using R software sva package, and finally, the data were analyzed using the statistical method Principal Component Analysis PCA [10] and visualization. See Fig 1a before batch correction and Fig 1b after batch correction. Cell senescence-related genes were obtained by searching and downloading from the CellAge database of cell senescence genes (The Database of Cell Senescence Genes: https://genomics.senescence.info/cells/) (SRG).

### 2.2 Methods

#### 2.2.1 Screening for differential senescence genes.

Differential expression analysis was performed on the combined dataset using the R package “limma, dplyr, pheatmap, ggplot2”, and differential genes (DEGs) were screened with the filtering conditions of corrected P.adj < 0.05 and |log2FC| ≥ 1.2. The heat map and volcano map of differential genes were drawn in R language for visualization. The heat map was shown as the 50 most significantly up-regulated differential genes and the 50 most significantly down-regulated differential genes. The volcano diagram was visualized with log2FC as the horizontal coordinate and -log10(adj.P.Val) as the vertical coordinate. The final senescence-related differential genes (hereinafter referred to as DESRGs) were obtained by intersecting the screened differential genes (DEGs) with the cellular senescence-related genes (SRGs) using the “VennDiagram” program package.

#### 2.2.2 Differential senescence gene GO functional enrichment analysis and KEGG pathway enrichment analysis.

Gene Ontology (GO) functional enrichment analysis divides gene function into three parts: cellular component (CC), molecular function (MF), and biological process (BP), which describes the cellular environment in which the gene product is likely to reside, the molecular function it performs, and the biological process it participates in, respectively [11]. Kyoto Encyclopedia of Genes and Genomes (KEGG) pathway enrichment analysis can systematically analyze the metabolic pathways of gene products in cells and the biological functions of gene products [12]. In this study, GO functional enrichment analysis and KEGG pathway enrichment analysis were performed using the R language clusterProfiler package, and visualized using the ggplot2 package. The GO analysis was set at P < 0.05, corrected P.adj < 0.05; and the KEGG analysis was set at P < 0.05.

#### 2.2.3 GSEA enrichment analysis.

Gene Set Enrichment Analysis GSEA (GSEA) is used to assess the distribution trend of genes of a predefined gene set in a table of genes sorted by phenotypic relevance, to determine their contribution to the phenotype [13]. In this study, samples were categorized into high and low-expression groups based on the median gene expression of the screened differential genes in the test and control groups. Using the “clusterProfiler” program package, the gene set c2.cp.kegg.Hs.symbols.gmt (MSigDB, http://www.gsea-msigdb.org/gsea/index.jsp) was selected as the reference gene set for enrichment analysis. The reference gene set was analyzed for enrichment, and the top 5 gene sets were filtered according to the threshold of P < 0.05, and the results were displayed.

#### 2.2.4 Protein-protein interaction networks (PPI).

The STRING database (https://cn.string-db.org/) was used to construct a protein-protein interaction (PPI) network for the intersected differentially senescent DESRGs, setting the condition as “Homo Sapiens Homo Sapiens”, set confidence > 0.4, hide single nodes that do not interact, and analyze the topology based on mediativity, tightness, and connectivity [14]. Screening ze key targets for AR treatment. PPI networks were visualized and analyzed using Cytoscape 3.8.2 (https://cytoscape.org/).

#### 2.2.5 Key modules and Hub gene screening.

The PPI network was weighted by the MCODE (Molecular Complex Detection) [15] plugin in Cytoscape 3.8.2 to predict the key molecular modules in the PPI network, in which the parameters were set as Degree Cutoff≥2, haircut≥0.2, Node Score Cutoff ≥ 0.2, K-core ≥ 2, Max Depth = 100. The topological network algorithm was utilized to assign values to each gene through the CytoHubba plugin and sorted to mine the Hub genes and sub-networks. The CytoHubba plugin provides 11 topological analysis algorithms, Stress, Betweenness, Radiality, Closeness, EcCentricity, BottleNeck, Degree, MNC, DMNC, and MCC. The extracted intersection genes calculated by 11 algorithms such as MCC, DMNC, MNC, Degree, and EPC were identified as Hub genes with reference to the research method of XUE [16] et al. Differential expression analysis of Hub genes was carried out using the program package “dplyr, pheatmap, ggplot2”, and volcano plots of Hub genes was plotted with the filtering conditions of corrected P.adj < 0.05 and |log2FC| ≥ 1.2, and volcano plots were plotted with log2FC as the horizontal coordinate and -log10 (|log2FC) and -log10 (|log2FC) as the horizontal coordinate. -log10(adj.P.Val) as vertical coordinate.

#### 2.2.6 Hub gene protein interaction network construction (GeneMANIA).

To explore Hub genes and their interacting proteins, the GeneMANIA database [17] (http://genemania.org/) was used for protein-protein interaction network (GeneMANIA) analysis and protein functional analysis. Inputting Hub gene name, gene species selecting human species for searching, protein-protein interaction network diagram was automatically constructed and functionally displayed. The more connecting lines between proteins in the network diagram indicate stronger interconnections.

#### 2.2.7 Predicting the Hub gene ceRNA regulatory network.

Firstly, miRanda, miRDB, miRWalk, and TargetScan target gene prediction databases were used to predict the differential miRNAs bound by Hub gene mRNAs, and the predicted differential miRNAs were taken as intersections with the mRNAs to construct mRNA-miRNA interaction pairs. The data were processed using Perl, and the filtering condition was miRNAs that were positive in all 4 databases. Next, the spongeScan database was used to predict lncRNAs interacting with differential miRNAs, and miRNA-lncRNA interaction pairs were obtained. Cytoscape 3.8.2 software was used to integrate and obtain the ceRNA network of mRNA-miNA-lncRNA interactions, which was visualized.

#### 2.2.8 Transcription factor regulatory networks.

To predict key transcription factors (TFs) of Hub genes, version 2 of the TRRUST database (https://www.grnpedia.org/trrust/) was used for predicting transcriptional regulatory networks. The TRRUST database contains databases of human and mouse transcriptional regulatory networks. The current version of the TRRUST database contains 800 human TFs and 828 mouse TFs with 8444 and 6552 regulatory relationships, respectively [18]. The transcription factors (TFs) regulating Hub genes were obtained from the TRRUST database, and the transcription factor regulatory network was visualized using Cytoscape software with P < 0.05 of FDR as the screening condition.

#### 2.2.9 Hub gene-drug interaction analysis.

The Drug-Gene Interaction database (DGIdb) is a database of drug-gene interactions that provides information on the association of genes with their known or potential drugs, containing information on more than 40,000 genes, more than 10,000 drugs, involving more than 100,000 drug-gene interactions, and classified into 42 drug classifications, and at least 49 interaction types defined by the source dataset, including inhibitors, activators, cofactors, ligands, and vaccines, which allow results to be screened by source, interaction type, or gene class [19]. In this study, we predicted Hub gene-drug interactions with the help of DGIdb database (https://dgidb.org/), downloaded the file “DSigDBv1.0 Detailed.txt”, and used the software R The “clusterProfiler, org.Hs.e.g.,db, enrichplot, ggplot2” package was used to analyze the Hub genes and construct the gene-drug interaction network with the filtering condition of P < 0.05 and corrected P.adj < 0.05 for the visualization. Visualization.

#### 2.2.10 Molecular docking.

SDF files were obtained from the Pubchem database (https://pubchem.ncbi.nlm.nih.gov/) by querying gene-drug interactions for screening to obtain the structures of key components of clinically used AR therapeutics [20]. The 3D structures of drug-enriched Hub gene core target proteins were downloaded from the PDB database (https://www.rcsb.org) and saved in PDB format [21]. Finally, molecular docking was performed using the CB-Dock2 (https://cadd.Labshare.co.uk/cb-dock2) online tool, and the lowest binding energy data were saved as the result of molecular docking [22]. The lower the binding energy, the stronger the binding of the active ingredient to the target protein, and it is generally believed that drug molecules with binding energies ≤ −5.0 kJ/moI have better binding activity to the target.

Ethical oversight and endorsement for this investigation were exempted, given the utilization of data from a public repository. Affirmative consent had been secured from all participants whose data were included in the public database study.

## 3. Results

### 3.1 Results of differential senescence gene screening

**Fig 2:**
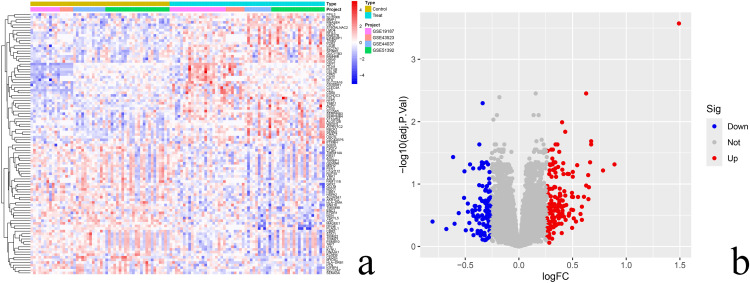
Differential expression landscape of senescence-related genes in allergic rhinitis. **(a)** Differential gene heat map: red indicatesup-regulation, blue indicates down-regulation, darker color represents higher differential gene expression, lighter color represents lower gene expression; **(b)** Differential gene volcano map: red indicates up-regulation, blue indicates down-regulation, grey indicates non-significant difference, the horizontal coordinate Log2FC value is the magnitude of the difference of the gene’s expression in the two samples, the bigger value represents the bigger difference, the vertical coordinate adj.P.Val value is the corrected P value, larger value represents higher confidence.

**Fig 3:**
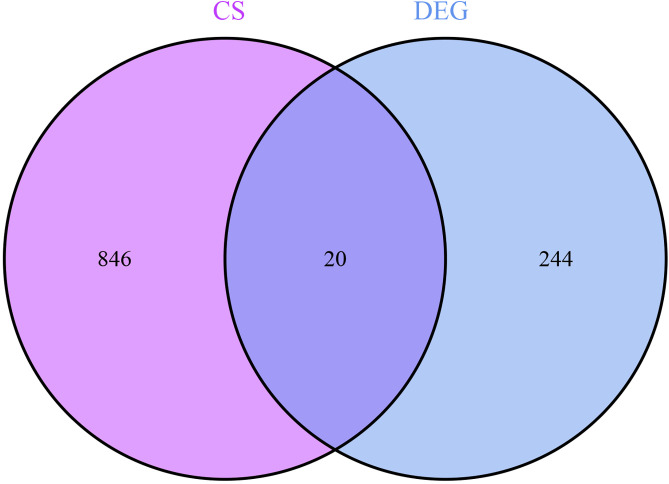
Intersection plot of differential and senescent genes.senescent genes are shown in purple and differential genes are shown in blue.

264 differential genes were obtained by screening the combined dataset with corrected P.adj < 0.05 and |log2FC| ≥ 1.2 as the filtering conditions, of which 107 were down-regulated genes and 157 were up-regulated genes. Differential gene heat map and volcano map were performed in R language, see Fig 2, a is the differential gene heat map, and b is the differential gene volcano map. 866 cellular senescence-related genes (SRGs) were obtained by cellular senescence genes. The intersection of 264 differential genes (DEGs) with 866 cellular senescence genes (SRGs) was taken to finally obtain 20 differential senescence genes (DESRGs) Fig 3.

### 3.2 GO and KEGG analysis of differential aging genes

**Fig 4:**
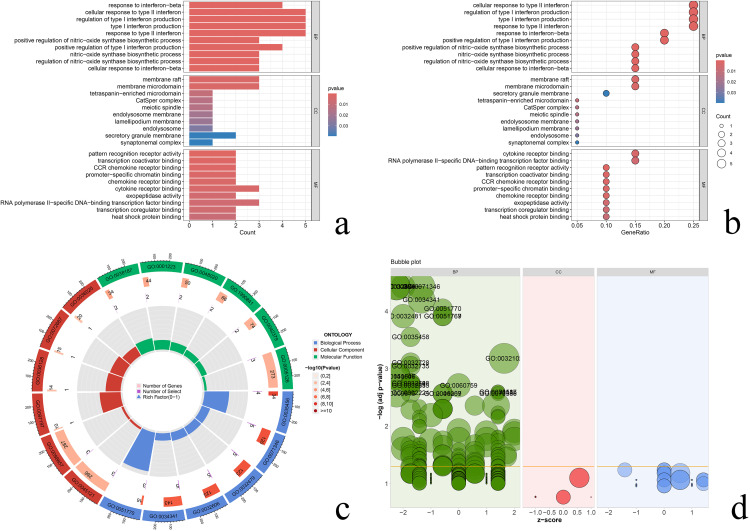
GO functional enrichment analysis. **(a)** bar chart, the length of the bar represents the number of genes enriched on the entry, the color from blue to red reflects the smaller P-value; **(b)** bubble chart, the size of the bubble reflects how many genes are enriched on the entry, the redder the color reflects the smaller the P-value; **(c)** circle chart, the outermost circle is the id of the GO, the 3 colors represent the 3 parts of the CC, MF, and BP; the 2nd circle is the number of genes distributed on the GO entry number of genes, the 3rd circle is the number of differential genes enriched on GO categories, and the innermost circle reflects the enrichment factor value; **(d)** GO classification bubble plot with z-score in the horizontal coordinate, vertical coordinate, and -log (adj p-value) in the vertical coordinate, and the higher vertical coordinate reflects the more significant gene enrichment.

GO functional enrichment analysis showed that differential senescence genes were enriched in 478 biological processes such as response to interferon-beta, cellular response to type II interferon, and regulation of type I interferon production (BP), enriched in 12 cellular components such as membrane raft, membrane microdomain, tetraspanin-enriched microdomain (CC), enriched in pattern recognition receptor activity, transcription coactivator binding, CCR chemokine receptor binding, and 58 molecular functions (MF). Bar graph reflecting the number of genes on the enrichment function (Fig 4a). Bubble plot, reflecting the proportion of genes on the enriched function (Fig 4b). Circle plot, reflecting the overall situation of genes enriched for GO (Fig 4c). bubble plot of GO classification, reflecting the significant situation of gene enrichment (Fig 4d).

**Fig 5:**
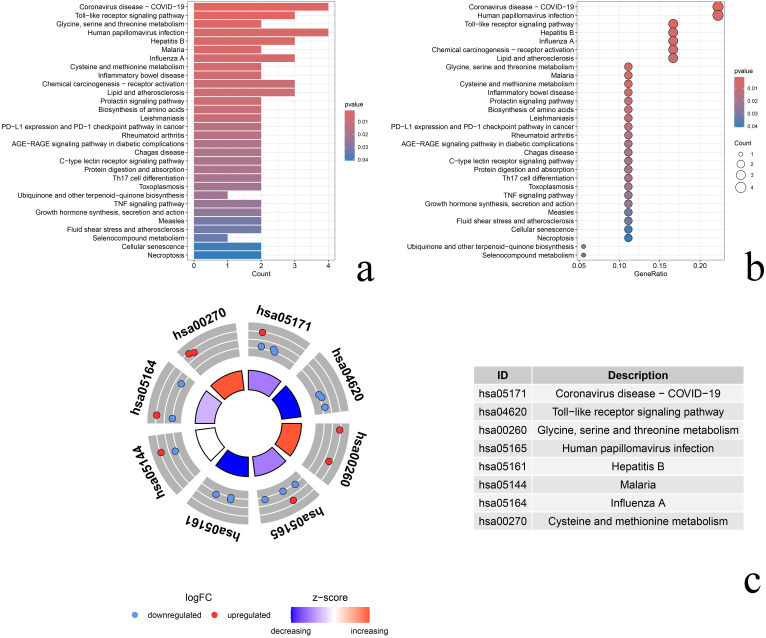
KEGG pathway enrichment analysis. **(a)** Bar graph, the length of the bar represents the number of genes enriched in the signaling pathway, the redder the color reflects the smaller the P-value; **(b)** Bubble graph, the size of the bubble reflects the proportion of genes enriched in the signaling pathway, the redder the color reflects the smaller the P-value; **(c)** Circle graph, the outermost circle is the id of the pathway; the second circle is the number of genes distributed in the pathway, the red color indicates up-regulation, and the blue color indicates down-regulation; the innermost circle reflects z-score; the innermost circle reflects the z-score.

KEGG pathway enrichment analysis showed that differential senescence genes were mainly enriched in 32 signaling pathways such as Coronavirus disease-COVID-19, Toll-like receptor signaling pathway, and Glycine, serine and threonine metabolism pathways. Bar graph reflecting the number of genes enriched in the signaling pathway (Fig 5a). Bubble plot, proportion of genes reflecting enrichment in signaling that pathway (Fig 5b). Circle plot, reflecting z-score values of genes on the pathway (Fig 5c).

### 3.3 Results of GSEA enrichment analysis

**Fig 6:**
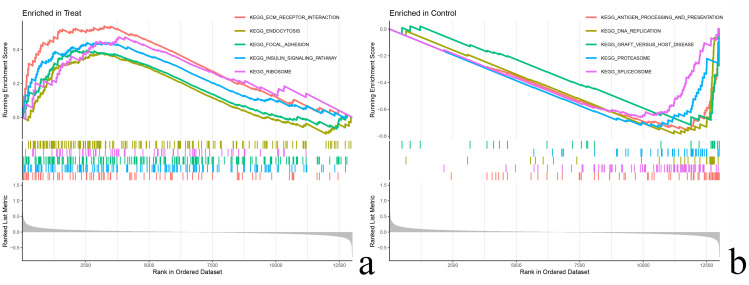
GSEA enrichment plots. **(a)** test group; **(b)** control group.The upper part of the graph represents the scores of the genes in the pathway enrichment, and the lower part of the graph represents the expression of the RANK values of the genes, with the left side representing high expression and the right side representing low expression.

The 264 differential genes were analyzed by the GSEA method using the R software package “limma, clusterProfiler, org.Hs.e.g.,db, enrichplot”, and the results of the GSEA enrichment analysis showed that the experimental group was mainly enriched in the following genes: ECM RECEPTOR INTERACTION, ENDOCYTOSIS, FOCAL ADHESION, INSULIN SIGNALING PATHWAY, and RIBOSOME pathways in the test group (Fig 6a), while the control group was mainly enriched in ANTIGEN PROCESSING AND PRESENTATION, DNA REPLICATION, GRAFT VERSUS HOST DISEASE, PROTEASOME, and SPLICEOSOME pathways (Fig 6b).

### 3.4 PPI network, key modules, and Hub gene screening results

**Fig 7:**
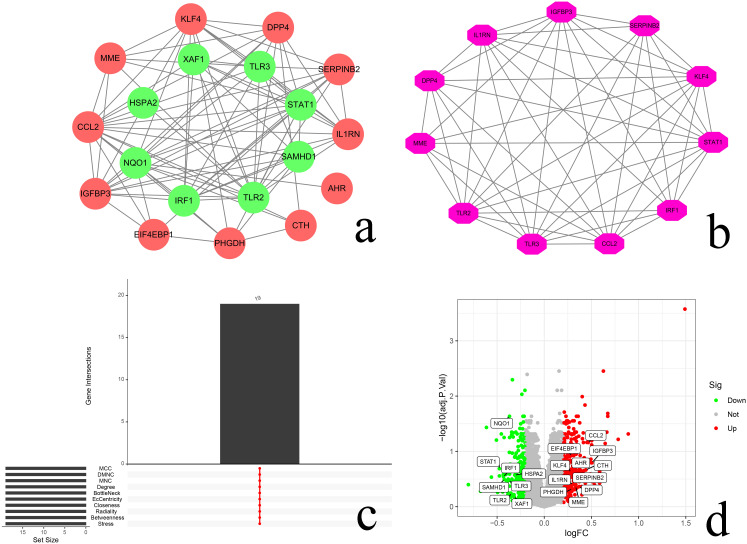
Shows the PPI network of 19 differentially senescent genes, where each node represents a protein, while each edge represents. **(a)** Protein-protein interaction. Red indicates up-regulated genes and blue indicates down-regulated genes; **(b)** For the MCODE cluster module score of 8.000, consisting of 11 nodes; **(c)** Plot of Hub gene scoring; **(d)**Volcano plot: for Hub gene expression, red indicates up-regulation, blue indicates down-regulation, and gray indicates non-significant difference, the horizontal coordinate Log2FC value is the size of the difference in the expression of the gene in the two samples, and the larger value represents the larger difference, the vertical coordinate adj.P.Val value is the corrected P value, and the larger value represents the larger confidence level.

The potential interactions between the above 20 differential senescence genes were searched through the STRING database, and the results showed that these genes have certain interactions in biological systems, obtaining 19 nodes and 86 edges consisting of 19 nodes and 86 edges, as shown in Fig 7a. One important gene cluster module was identified through MCODE plugin, which was composed of 11 nodes with the highest clustering score (score: 8.000), Fig 7b. through the CytoHubba plugin’s will ClusteringCoefficient, Stress, Betweenness, Radiality, Closeness, EcCentricity, BottleNeck, Degree, MNC, DMNC, and MCC calculated by a total of 11 algorithms to identify the extracted intersecting genes as Hub genes, and finally identified 19 overlapping Hub genes (CCL2, STAT1, TLR2, IGFBP3, TLR3, KLF4, IL1RN, IRF1, SERPINB2, DPP4, MME, NQO1, SAMHD1, XAF1, PHGDH, EIF4EBP1, CTH, HSPA2, AHR) as shown in Fig 7c. The expression of 19 Hub genes was visualized by “Dplyr, ggplot2, ggrepel”, with the filtering conditions of corrected P.adj < 0.05 and |log2FC| ≥ 1.2. The results were shown as up-regulated expression in CCL2, EIF4EBP1, IGFBP3, AHR, KLF4, CTH, SERPINB2, IL1RN, DPP4, PHGDH, and MME tissues, and down-regulated expression in AR tissues for NQO1, STAT1, IRF1, HSPA2, TLR3, SAMHD1, TLR2, and XAF1 (Fig 7d).

### 3.5 Results of Hub gene protein interactions network construction

**Fig 8:**
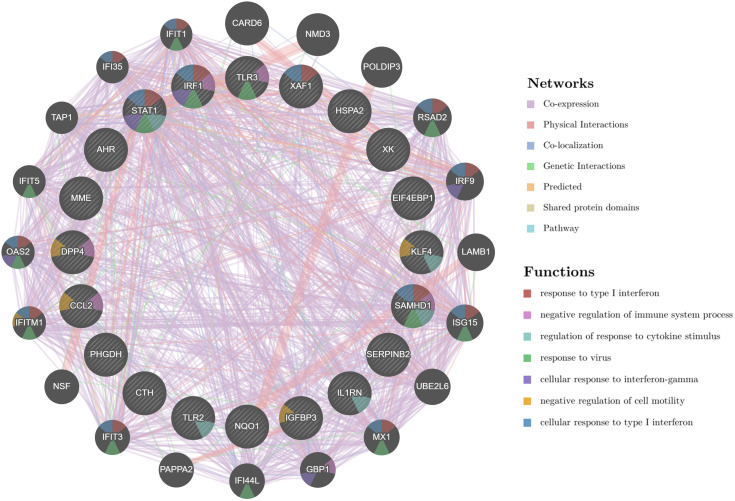
Hub gene GeneMANIA network diagram. Circle icons represent protein genes, the connecting lines between two circles represent the existence of interactions between two targets, the lines of different colors represent different interactions, and the colors within the circles represent different functions of the genes.

The 19 Hub genes were analyzed by GeneMANIA database to obtain the PPI network map (Fig 8). The map consists of 40 nodes, and the seven colored lines represent Co-expression, Physical Interactions, Co-localization, Genetic, Interactions, Predicted, and Shared protein domains, respectively, There are 21 functional proteins associated with CCL2, STAT1, TLR2, IGFBP3, TLR3, KLF4, IL1RN, IRF1, SERPINB2, DPP4, MME, NQO1, XK, SAMHD1, XAF1, PHGDH, EIF4EBP1, CTH, HSPA2 and AHR. CARD6, NMD3, POLDIP3, and RSAD2 were the most strongly associated. Different colors within the nodes represent different functional regulation, such as response to type I interferon, negative regulation of immune system process, regulation of response to cytokine stimulus, response to virus, cellular response to interferon-gamma, negative regulation of cell motility, cellular response to type I interferon, as shown in Fig 8.

### 3.6 ceRNA network construction

**Fig 9:**
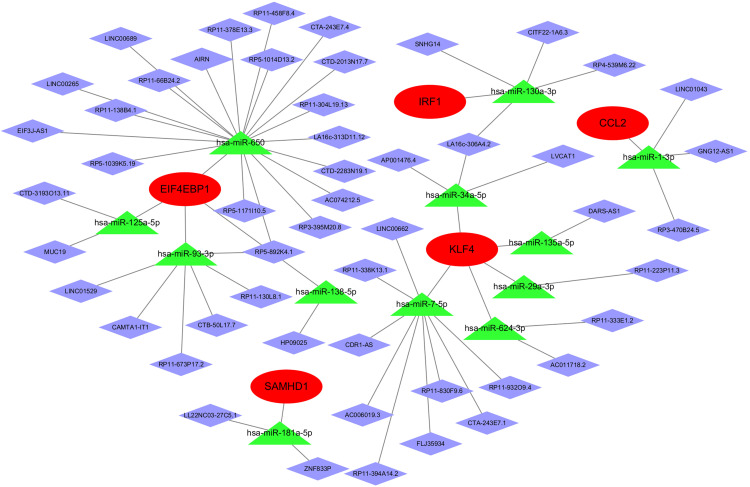
Shows the construction of ceRNA network. Red is Hub gene mRNA, green represents miRNA, purple diamond represents lncRNA, each connecting line represents the interactions between mRNA and miRNA, miRNA and lncRNA.

The miRNAs that interact with Hub gene mRNAs were predicted by miRanda, miRDB, miRWalk, and TargetScan target gene prediction databases, and the prediction results were intersected with the differential miRNAs by taking the intersection of the predicted results to get 29 interactions. spongeScan database was used to predict the interactions with the differential miRNAs of the lncRNAs and take the intersection of the predicted result miRNAs with the differential lncRNAs to get 53 interacting lncRNAs. miRNAs and mRNAs that are not in this interaction relationship are deleted, and the integration results in a ceRNA network of mRNA-miRNA-lncRNA interactions. Fig 9, red oval nodes represent key gene mRNAs, green triangle nodes represent miRNAs, and purple diamonds represent lncRNAs. The network has 48 nodes (5 mRNA, 12 miRNA, and 41 lncRNA nodes) and 65 edges, and each edge represents the interworking relationship between mRNAs and miRNAs, and miRNAs and lncRNAs.

### 3.7 Transcription factor regulatory network

**Fig 10:**
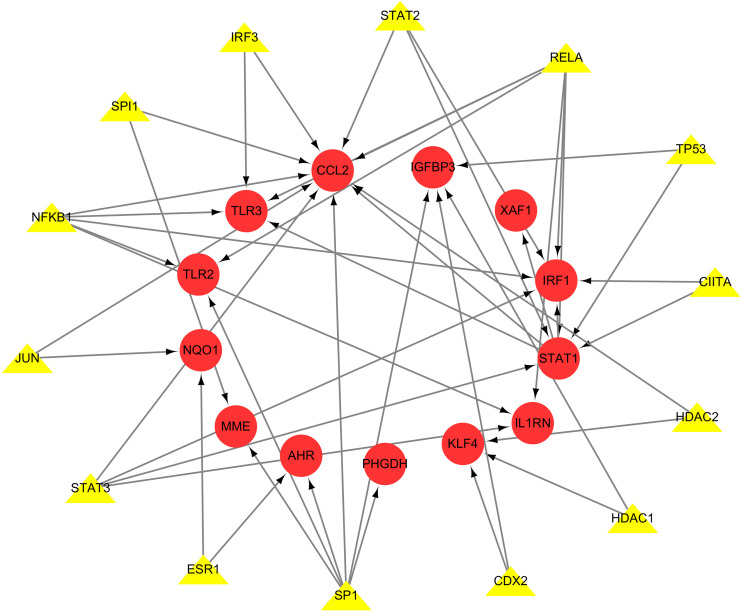
Transcription factor regulatory network diagram. Red circles are Hub genes, yellow triangles represent TFs, and each connecting line represents the interactions between Hub genes and TFs.

Based on the TRRUST database, 15 transcription factors regulating Hub genes were identified (Table 1), namely CDX2, CIITA, ESR1, HDAC1, HDAC2, IRF3, JUN, NFKB1, RELA, SP1, SPI1, STAT1, STAT2, STAT3, TP53, which are synergistically involved in IRF1, CCL2, STAT1, IL1RN, TLR2, TLR3, XAF1, MME, PHGDH, AHR, IGFBP3, KLF4, and NQO1, which are the 13 Hub genes, were regulated, see Fig 10.

**Table 1:** Hub gene transcription factors.

Key TF	Description	List of overlapped genes	Overlapped genes	P value	Q value
CDX2	caudal type homeobox 2	KLF4,IGFBP3	2	0.000468	0.000702
CIITA	class II, major histocompatibility complex, transactivator	IRF1,STAT1	2	0.000439	0.000702
ESR1	estrogen receptor 1	NQO1,AHR	2	0.00262	0.00302
HDAC1	histone deacetylase 1	KLF4,IGFBP3	2	0.00229	0.00286
HDAC2	histone deacetylase 2	CCL2,KLF4	2	0.000332	0.000623
IRF3	interferon regulatory factor 3	TLR3,CCL2	2	1.00E-04	0.000214
JUN	jun proto-oncogene	CCL2,NQO1	2	0.0097	0.0104
NFKB1	nuclear factor of kappa light polypeptide gene enhancer in B-cells 1	IRF1,TLR3,IL1RN,TLR2,CCL2	5	9.97E-06	2.73E-05
RELA	v-rel reticuloendotheliosis viral oncogene homolog A (avian)	IL1RN,CCL2,TLR2,TLR3,IRF1,STAT1	6	3.56E-07	2.67E-06
SP1	Sp1 transcription factor	TLR2,CCL2,MME,PHGDH,AHR,IGFBP3	6	4.87E-06	1.83E-05
SPI1	spleen focus forming virus (SFFV) proviral integration oncogene spi1	CCL2,MME	2	0.00175	0.00239
STAT1	signal transducer and activator of transcription 1, 91kDa	IRF1,XAF1,CCL2,TLR3	4	1.34E-06	6.72E-06
STAT2	signal transducer and activator of transcription 2, 113kDa	IRF1,CCL2,STAT1	3	1.42E-07	2.13E-06
STAT3	signal transducer and activator of transcription 3 (acute-phase response factor)	IL1RN,IRF1,STAT1,CCL2	4	1.09E-05	2.73E-05
TP53	tumor protein p53	STAT1,IGFBP3	2	0.0117	0.0117

### 3.8 Hub gene-drug interaction analysis

**Fig 11:**
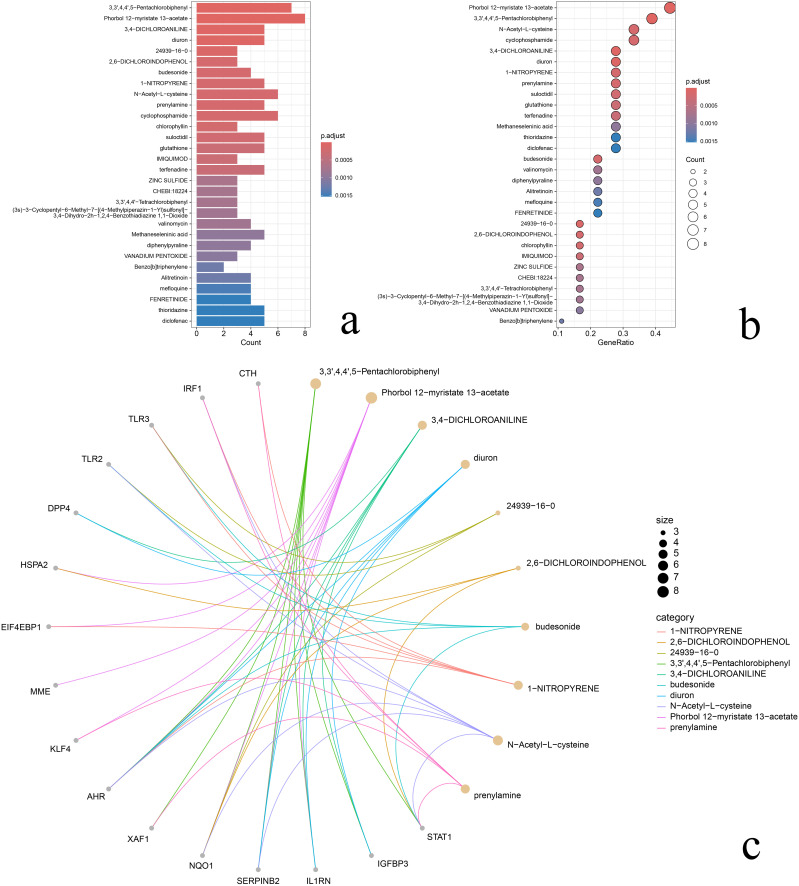
Gene-drug interactions analysis. **(a)** bar graph, the length of the column represents the number of genes enriched on the drug, and the color from blue to red reflects the smaller P-value; **(b)** bubble graph, the size of the bubbles reflects how many genes are enriched on the drug, and the redder the color reflects the smaller the P-value; **(c)** String graph, the circle represents the node, the grey circle represents the Hub gene, and the rest represents the drug, and the size of the drug node reflects how many genes are enriched, the larger the node the more genes are enriched, and each connecting line represents the interactions between the Hub gene and the drug. How many, the larger the node the more genes enriched, each connecting line represents the interactions between the Hub gene and the drug.

The results showed that there were 489 drugs that interacted with Hub gene and the top 10 drugs with significant enrichment were 3,3’,4,4’,5-Pentachlorobiphenyl, Phorbol 12-myristate 13-acetate, 3,4-DICHLOROANILINE, diuron, 24939-16-0, 2,6-DICHLOROINDOPHENOL, budesonide, 1-NITROPYRENE, N-Acetyl-L-cysteine, and prenylamine. All of these screened drugs have been associated with the regulation of Hub gene function, and their discovery provides more future prevention and treatment options for AR. Options. Bar graph reflecting the number of genes enriched on the drug (Fig 11a). Bubble plot with bubble size reflecting the proportion of genes enriched on the drug (Fig 11b). Chord plot, reflecting the interactions between Hub genes and the drug (Fig 11c).

### 3.9 Molecular docking

Budesonide nasal spray is the first-line medication for allergic rhinitis and is also the most effective drug. It cannot only continuously control the symptoms of rhinitis but also improve the quality of life and enhance sleep quality. Mometasone is a second-generation, highly potent nasal glucocorticoid with strong receptor affinity and pronounced local anti-inflammatory effects. It can rapidly alleviate symptoms such as itching, sneezing, rhinorrhea, and nasal congestion, while also improving olfactory function and sleep quality. Accordingly, it is recommended as a first-line treatment in current clinical guidelines.

**Fig 12:**
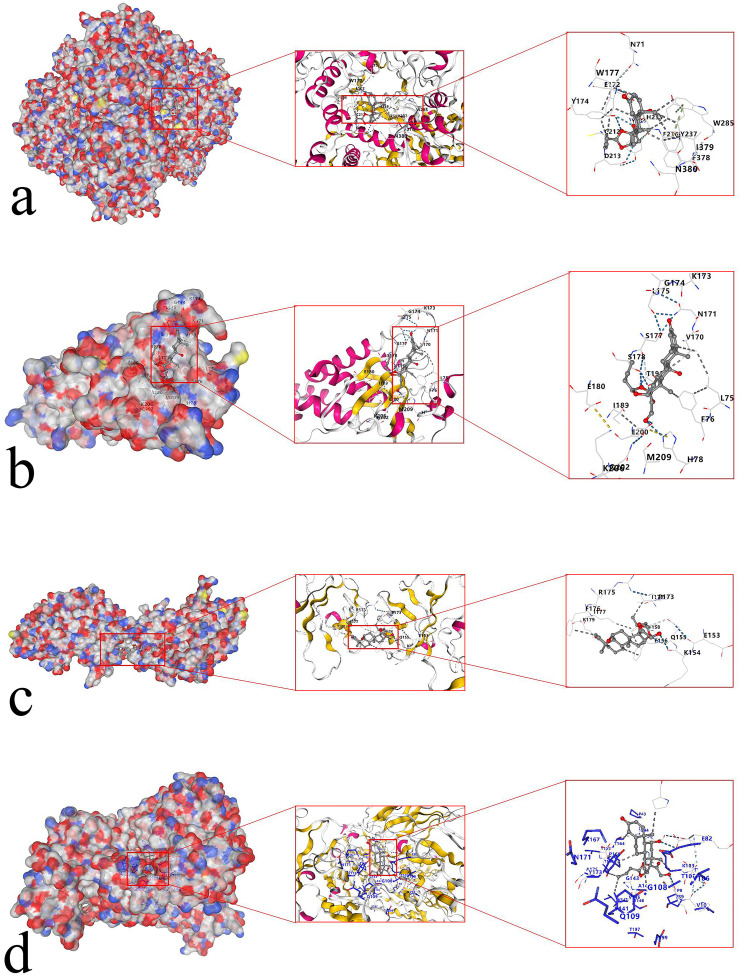
Schematic diagram of molecular docking of key target proteins for AR treatment with budesonide. **(a)** Budesonide docked to AHR protein; **(b)** Budesonide docked to STAT1 protein; **(c)** Budesonide docked to TLR2 protein; **(d)** Budesonide docked to TLR3 protein.

**Fig 13:**
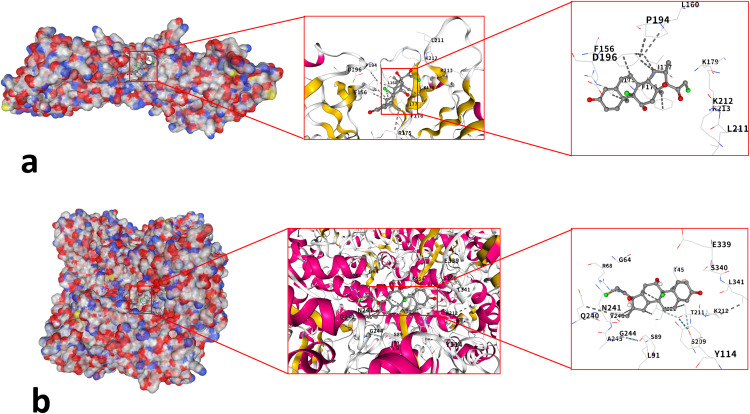
Molecular docking schematic diagram of the key target proteins of momezon in the treatment of AR. **(a)** Momezon docking with TLR2 protein; **(b)** Momezon docking with the CTH protein.

Through the network construction and screening of gene drugs, it was found that budesonide has regulatory effects on Hub genes STAT1, TLR2, TLR3, and AHR. Mometasone regulates the hub genes TLR2 and CTH. The primary chemical components of budesonide and mometasone, along with the key target proteins of the identified hub genes, were further validated through molecular docking. The results showed that the Vina score of budesonide for AHR was −9.0 (Fig 12a), the Vina score of STAT1 was −7.2 (Fig 12b); the Vina score of TLR2 was −6.9 (Fig 12c); the Vina score of TLR3 was −8.2 (Fig 12d). The Vina score of TLR2 of mometasone was −7.5 (Fig 13a); the Vina score of CTH was −7.3 (Fig 13b).

## 4. Discussion

Allergic rhinitis (AR) is a prevalent chronic respiratory disease that seriously affects the quality of life of patients. A study has shown that the incidence of allergic rhinitis is increasing year by year with accelerated industrialization, increased environmental pollution, and lifestyle changes, and the patient population is becoming younger, with a significant impact on the well-being of millions of people [23]. However, despite great efforts, the pathogenesis of AR is not yet fully understood clinically. Currently, the main treatments for allergic rhinitis include pharmacotherapy, immunotherapy, and lifestyle modification; however, most of these pharmacological therapies can only alleviate the symptoms but not cure the disease [24]. Therefore, there is an urgent need to explore new biomarkers of diagnostic and prognostic significance and to understand the etiology and molecular mechanisms of AR to establish effective treatments and improve its prognosis.

Cellular senescence is an organism’s self-protective mechanism in response to various stressful conditions, but excessive cellular senescence can accelerate the disease process [25]. In recent years, the rapid development of genetic technologies and bioinformatics analysis of cellular senescence has made it possible to find potential targets by exploring the integration of information on AR diagnosis, treatment, and prognosis. In this study, data were obtained from GSE19187, GSE43523, GSE44037, and GSE51392 through GEO, which included 58 AR samples and 52 samples from healthy controls. Comprehensive analysis by nasal mucosa tissue microarray from AR patients yielded 264 differential genes (DEGs) with 866 cellular senescence genes (SRGs), and a total of 20 differential senescence genes (DESRGs) were screened by taking the intersection. The 19 hub genes are quantifiable in nasal epithelial brushes, providing a senescence-associated molecular signature that may refine current IgE/FeNO-based stratification of refractory allergic rhinitis (AR). Protein–protein interaction (PPI) network analysis of differentially expressed senescence-related genes (DESRGs) identified these hub genes, including CCL2, EIF4EBP1, IGFBP3, AHR, CTH, SERPINB2, IL1RN, DPP4, PHGDH, MME, and KLF4 as upregulated, and NQO1, STAT1, IRF1, HSPA2, TLR3, SAMHD1, TLR2, and XAF1 as downregulated. These genes exhibit high topological connectivity and centrality within the network, highlighting their potential roles as key regulatory nodes in AR pathogenesis. Integrative analyses further revealed their involvement across multiple regulatory layers. ceRNA network analysis suggested that five hub genes (e.g., STAT1, TLR2, AHR) are potentially regulated by 29 miRNAs and 53 lncRNAs. Transcription factor (TF) network analysis indicated that 15 TFs, including STAT1, NFKB1, and RELA, may regulate hub gene expression. Drug–gene interaction analysis identified 489 compounds targeting these hub genes. Among these, budesonide interacts with STAT1, TLR2, TLR3, and AHR, while mometasone interacts with TLR2 and CTH; these interactions were further validated by molecular docking. Notably, IGFBP3, SERPINB2, AHR, NQO1, and STAT1 exhibited the strongest drug-enrichment signals. Budesonide and mometasone are widely used nasal corticosteroids with potent anti-inflammatory effects. Budesonide shows high affinity for STAT1, TLR2, TLR3, and AHR, whereas mometasone targets TLR2 and CTH. Optimizing these drugs based on their molecular structures may enhance anti-inflammatory efficacy and potentially counteract senescence-related mechanisms in AR. Additionally, 485 DGIdb-curated compounds, including N-acetylcysteine and imiquimod, provide an experimentally accessible repertoire for ex vivo organoid or in vivo validation. Conversely, xenobiotics predicted to upregulate pro-senescence hub genes, such as diuron, could be prioritized for avoidance in pharmacovigilance. Finally, the miR-146a-5p–SERPINB2 ceRNA axis is detectable in nasal exosomes, offering a non-invasive liquid biomarker to monitor therapeutic response or impending relapse in future clinical trials. Collectively, these findings highlight the 19 hub genes as promising biomarkers and therapeutic targets, whose dysregulation may contribute to AR development through perturbations in immune responses, inflammatory signaling, and cellular senescence pathways. Experimental validation remains essential to confirm these computational predictions, with IGFBP3, SERPINB2, AHR, NQO1, and STAT1 prioritized for further investigation.

IGFBP3 is one of the most abundant IGF-binding proteins in the blood after birth. It binds to IGFs, prevents them from being degraded, facilitates the transport of IGFs in various parts of the body, and modifies the structure of IGFs thereby altering the interaction between IGFs and their specific receptors [26]. At the same time, IGFBPs have antiproliferative, anti-mitotic, and pro-apoptotic effects [27]. Veraldi et al [28]. showed that baseline cell-associated IGFBP3 levels in airway epithelial cells of patients with allergic asthma were higher than those of healthy donors, and that these levels increased significantly with disease severity and deterioration of lung function, which could be used as a marker of the inflammatory response in asthma. Interestingly, eosinophils can influence airway remodeling through TGF-β directly or indirectly by inducing IGFBP3 expression in subepithelial fibroblasts [28]. In this study, we found that IGFBP3 could participate in the negative regulation of cell motility through gene-protein interaction network construction. Four transcription factors regulating IGFBP3 were found to be SP1, CDX2, HDAC1, and TP53, and a total of 47 drugs interacting with IGFBP3 were identified, such as 3,4-DICHLOROANILINE, Diuron, Cyclophosphamide, Chlorophyllin, Diclofenac, and so on. Serine protease inhibitor B2 (SerpinB2) is a member of the serine protease inhibitor B branch or ovalbumin-like serine protease inhibitor subgroup. Zhao et al [29]. demonstrated that SERPINB2 is highly expressed in nasal brushes of patients with AR and plays a key role in Th2-mediated immune responses. Meanwhile, SERPINB2 has been shown to correlate with FEV1, FeNO, peripheral and sputum eosinophils and asthma severity, and has been identified as a biomarker of T2 inflammation in asthma [30]. In a controlled experiment, SERPINB2 was found to be increased in eosinophilic chronic rhinosinusitis with nasal polyps (CRSwNP) nasal polyp epithelial cells and to promote T2 inflammation through STAT6 signaling, which could be considered a novel therapeutic target for CRSwNP. Studies have shown that the miR-146a-5p/SERPINB2 pathway can inhibit the differentiation of Th2 cells [31]. Zhou et al [32]. found that miR-146a-5p expression was negatively correlated with the expression of SERPINB2 in the sera of AR patients, which could be used as a potential target for the treatment of AR through the miR-146a-5p/ SERPINB2 signaling pathway. In this study, a total of 80 drugs were found to interact with SERPINB2, such as 3,3’,4,4’,5-Pentachlorobiphenyl, Phorbol 12-myristate 13-acetate, 3,4-DICHLOROANILINE, Diuron, Glutathione, etc. The aryl hydrocarbon receptor (AHR) belongs to PAS (Per-ARNT-Sim), a superfamily of transcription factors widely present in many tissues throughout the body [33]. It has been found that AHR sensing and response to environmental stimuli can play an important role in cellular developmental processes and immune regulation [34]. At the same time, AHR plays a regulatory role in mediating innate and adaptive immune responses against various infectious diseases, metabolic disorders, cancers, and allergic diseases [35]. In a randomized controlled trial, AHR was demonstrated to be one of the potential mechanisms underlying the Th17 response in the pathogenesis of AR, with increased levels of AHR expression in patients with AR and a strong correlation with clinical severity [36]. Riaz et al [37]. reviewed AHR and found that AHR plays an important role in allergic diseases and has a significant impact on the expression and control of mast cells, B-cells, macrophages, antigen-presenting cells (APCs), the Th1/ Th2 cell balance, and Th17. Th2 cell balance, Th17, and regulatory T cells. In this study, two transcription factors regulating AHR were identified as SP1 and ESR1, and a total of 244 drugs interacting with AHR were identified, such as Budesonide, Diuron, IMIQUIMOD, Diphenylpyraline, Isorhamnetin, and so on.

Quinone oxidoreductase (NQO1), encoded by the NQO1 gene, prevents oxidative damage to DNA caused by environmental stressors, maintains the reduced forms of ubiquinone and α-tocopherol quinone, and plays an important role in the protection of endogenous antioxidants [38]. Lee,et al [39]. It was found that the production of reactive oxygen species (ROS) was reduced in fibroblasts through the NQO1 pathway, and the treatment abrogated the inflammation-induced increase of IL-6 and IL-8 levels in nasal fibroblasts. Pyun et al [40]. showed that the NQO1 antioxidant enzyme promotes ROS detoxification and serves as a biomarker of oxidative stress, but regulation of antioxidant activity and ROS production is required to treat AR. In this study, we identified 2 transcription factors that regulate NQO1 as JUN and ESR1, and a total of 102 drugs that have interactions with AHR, such as Chlorophyllin, Glutathione, Methaneseleninic acid, OZONE, and Dichloromercury. The signal transducer and activator of transcription 1 (STAT1) is an important protein that links signaling between receptors and effectors in the cell membrane.STAT1 is mainly activated by type I and type II interferons, which are often considered antagonists of Th2 development and allergic inflammation [41]. Gernez et al [42],studies have found that the presence of STAT1 is negatively correlated with total serum IgE levels in German children and that STAT1 has an anti-allergic function. Zhou et al [43]. induced the production of CD4 + CD25 + FoxP3 + Treg cells in the IL-35/STAT1 pathway in AR by taurine, restored Treg populations to normalize inflammatory responses, alleviated the symptoms of AR and reduced the histopathological signs of AR. Reduce histopathologic signs of AR. In Hattori’s study, STATI1 mice exhibited impaired nasal eosinophilia and significantly reduced histamine-induced nasal hyperresponsiveness after sensitization to SEA. Lei et al [44]. found that STAT1 expression was down-regulated in AR patients compared to normal controls. In this study, gene-protein interaction network construction revealed that STAT1 can participate in response to type I interferon, regulation of response to cytokine stimulus, response to virus, cellular response to interferon-gamma, and cellular response to type I interferon. And 5 transcription factors regulating STAT1 were found to be STAT2, RELA, STAT3, CIITA, and TP53. 77 drugs with interaction with STAT1 were found, such as Budesonide, N-Acetyl-L-cysteine, Prenylamine, Chlorophyllin, and Suloctidil.Although this study systematically identified hub genes associated with cellular senescence in allergic rhinitis (AR) using integrated bioinformatics approaches, several limitations should be acknowledged. First, all findings are based on computational analyses of publicly available datasets, without experimental validation; thus, the expression patterns and functional roles of the identified hub genes require confirmation through in vitro and in vivo studies. Second, the absence of clinical samples and patient-level data precluded correlation of gene expression with disease severity, treatment response, or long-term prognosis. Third, potential confounding factors—such as age, sex, comorbidities, and environmental exposures—were not accounted for, which may affect gene expression profiles and limit the generalizability of the results. Finally, although ceRNA and transcription factor regulatory networks were constructed, these predictions rely on computational algorithms and warrant further experimental validation. Future studies should integrate multicenter clinical cohorts with functional experiments to enhance the robustness, reproducibility, and translational potential of these findings.

## 5. Conclusion

In summary, in this study, we screened 19 Hub genes associated with AR cellular senescence by bioinformatics analysis and constructed a PPI network, a ceRNA network, a transcription factor regulatory network and a gene-drug interaction network. These findings not only provide new insights into the pathogenesis of AR but also provide new targets and ideas for the diagnosis and treatment of AR. Future studies should further validate the function and clinical application value of these Hub genes with the aim of developing more effective AR treatments.

## Supporting information

S1 File Human subjects research checklist. (DOCX)
