# Supplementary material for: Mechanisms of cellular senescence combined with molecular docking strategies: A biomarker study of potential therapeutic targets for allergic rhinitis
Source: PLoS One. 2026 Jan 9;21(1):e0338309. doi: 10.1371/journal.pone.0338309 (PMC12788676; doi:10.1371/journal.pone.0338309)
Supplement: S1 File — (DOCX) [file pone.0338309.s001.docx]

**Human Participants Research Checklist**

***Complete the following if your study involved human participants or human participants’ data. These questions should be addressed for prospective and retrospective studies.***

1. Did you obtain ethics approval for this study?
   - If yes, please upload (file type “Other”) all the approval documents you received from your ethics committee to cover the entire range of the study period (i.e. the original approval document and any extension documents). Where ethics approval was obtained from more than one study location, please provide approval document(s) from all of the sites. If the original document is in another language, please also provide an English translation.

___ Uploaded _√__ N/A

- - If you did not obtain ethical approval, please explain why this was not required below.

Ethical oversight and endorsement for this investigation were exempted, given the utilization of data from a public repository. Affirmative consent had been secured from all participants whose data were included in the public database study.

1. If you prospectively recruited human participants for the study – for example, you conducted a clinical trial, distributed questionnaires, or obtained tissues, data or samples for the purposes of this study, please report in the Methods:
   1. the day, month and year of the **start and end** of the recruitment period for this study.
   2. whether participants provided informed consent, and if so, what type was obtained (for instance, written or verbal, and if verbal, how it was documented and witnessed). If your study included minors, state whether you obtained consent from parents or guardians. If the need for consent was waived by the ethics committee, please include this information.

Please state the line number(s) in the Methods where this is reported

_Four AR-related gene chips (GSE19187, GSE43523, GSE44037, and GSE51392) were downloaded from the gene expression database (GEO) for data pooling. Screening differential genes (DEGs) were taken to intersect with cellular senescence-related genes (SRGs) to obtain differential senescence genes (DESRGs). The differential senescence genes were subjected to Gene Ontology Database (GO) functional analysis, Kyoto Encyclopedia of Genes and Genomes (KEGG) pathway analysis, and GSEA enrichment analysis. Protein-protein interaction (PPI) networks were constructed through the STRING database, MCODE plugin weights were analyzed to identify important gene cluster modules, and Hub genes were screened using the CytoHubba plugin topological network algorithm. Hub gene protein interactions network (GeneMANIA) was constructed by the GeneMANIA database. Predict Hub gene construct mRNA-miNA-lncRNA interactions by miRanda, miRDB, miRWalk, TargetScan, and spongeScan databases; construct Hub gene transcription factor regulatory networks by TRRUST database; analyze Hub gene-drug interactions by DGIdb database and select commonly used drugs in the clinic for molecular docking validation.

___ Completed __√_ N/A

1. If you are reporting a retrospective study of, for example, medical records, archived samples, survey data, please report in the Methods section:
2. the day, month and year when the data were accessed for research purposes
3. whether authors had access to information that could identify individual participants during or after data collection

Please state the line number(s) in the Methods where this is reported __The datasets presented in this study can be found in online repositories. The names of the repository/repositories and accession number(s) can be found in the article/Supplementary Material.___ Completed ___ N/A
